# Novel box trainer for taTME – prospective evaluation among medical students

**DOI:** 10.1515/iss-2019-0013

**Published:** 2019-10-07

**Authors:** Jakob Mann, Jens Rolinger, Steffen Axt, Andreas Kirschniak, Peter Wilhelm

**Affiliations:** Department of Orthopaedic and Trauma Surgery, DIAKOVERE Henriettenstift Hospital, Hannover, Germany; Department of Surgery and Transplantation, University of Tuebingen, Tuebingen, Germany

**Keywords:** box trainer, surgical training, taTME, transanal rectal resection

## Abstract

**Background:**

Transanal total mesorectal excision (taTME) has been subject to extensive research and increasing clinical application. It allows further reduction of trauma by accessing via a natural orifice. Manifold platforms and instruments have been introduced and heterogeneity in surgical techniques exists. Because of the technique’s complexity there is a persistent need for dedicated training devices and concepts.

**Materials and methods:**

The key steps of taTME were analyzed and a box trainer with three modules resembling these steps was designed and manufactured. Twenty-one surgically inexperienced medical students performed five repetitions of the three tasks with the new box trainer. Time and error count were analyzed for assessment of a learning curve.

**Results:**

A significant reduction of processing time could be demonstrated for tasks 1–3 (p < 0.001; p < 0.001; p = 0.001). The effect size was high for comparison of repetition 1 and 5 and decreased over the course (task 1: r = 0.88 vs. r = 0.21; task 2: r = 0.86 vs. r = 0.23; task 3: r = 0.74 vs. r = 0.44). Also, a significant reduction of errors was demonstrated for tasks 1 and 2. The decrease of effect size was analogously demonstrated.

**Conclusions:**

The trainer might help to reduce the use of animal models for testing of platforms and instruments as well as gaining first-hand experience in transanal rectal resection.

## Introduction

During the 1980s the transanal access was significantly promoted by the introduction of the transanal endoscopic microsurgery (TEM) procedure by Buess et al. [1], [2], [3]. Initially limited to a very selected cohort of patients [4], [5], [6], this access has been subject to increasing attention and research as a platform for rectal resection and transanal total mesorectal excision (taTME) in terms of a natural orifice transluminal endoscopic surgery (NOTES) since the late 2000s [7], [8], [9], [10], [11], [12], [13], [14], [15]. While a low anterior resection [16] with TME remains the gold standard for oncologic therapy of mid and low rectal carcinoma [4], ongoing data indicate a possible benefit for a down-to-up execution in terms of taTME [17], [18], [19]. Nearly all published experimental data and all clinical case series covering the application of taTME must be referred to as hybrid-taTME [18], [19], [20]. The technique described by this term is composed of a primary laparoscopic preparation with secondary transanal access or simultaneous preparation in terms of a two-team-approach. Experimental data and clinical case reports of merely transanal execution (pure taTME) are scarce [16], [21], [22], [23]. Presumed benefits of a down-to-up approach are excellent view on the operational field and, hence, more detailed preparation within the narrow pelvis [17]. Disadvantages of the transanal approach have been described since its introduction: in comparison to laparoscopy triangulation is drastically reduced, subsequently, degrees of freedom are limited, and delimitation of movements prolongate the learning curve [5], [23]. Novel port systems have partially managed to overcome these obstacles [23], [24], [25]. Especially dedicated single-use instruments made taTME more accessible for wider use [24], [25]. Yet, high complexity of transanal surgical access is associated with a significant learning curve. A reduction in the learning curve might be enabled by implementation of standardized institutional operative protocols together with proficient proctorship, but data is limited [26].

Dedicated training models for transanal access are scarce and existing training scenarios widely use human cadavers or animal models, which raise ethical, infrastructural, and financial considerations. In the context of new innovative platforms there seems to be a necessity for the evaluation of such devices outside of university infrastructures and animal models. Assessment of operation platforms should be the first approach to new operational access routes before implementation into clinical practice. We present a dedicated box trainer for taTME and its evaluation by a prospective study among medical students.

## Materials and methods

### The Tuebinger taTME trainer (taTME-trainer)

During preliminary studies [7], [8], [23], we identified surgical key steps for taTME of which the following qualified for reproduction and abstraction:

1. closure of the rectal lumen by application of a purse string suture (PSS),
2. circular endoluminal marking of the transsection line,
3. circular preparation and transection.

**Figure 1:**
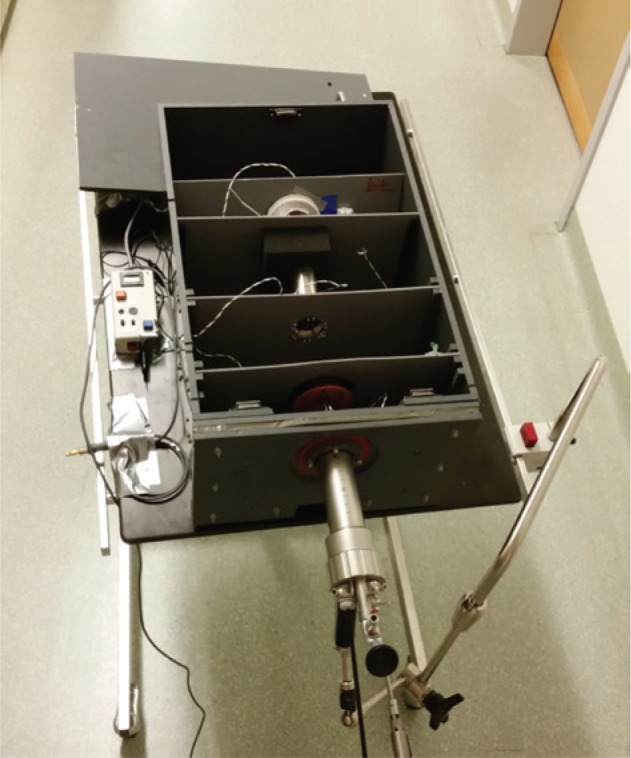
The uncovered box device with the TEO^®^ system (Karl Storz GmbH & Co. KG, Tuttlingen, Germany) introduced.

**Figure 2:**
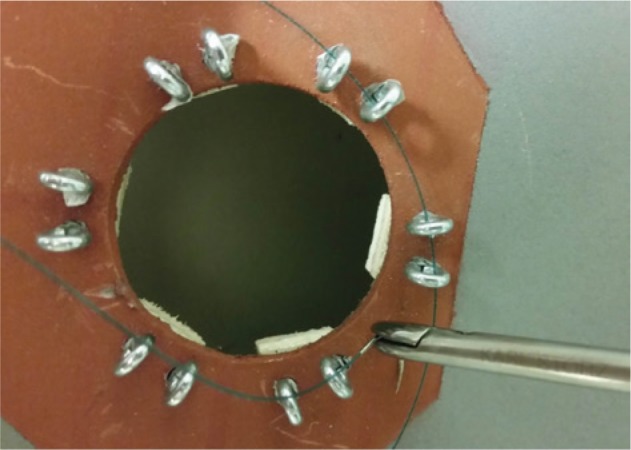
PSS module with steel loops at a third completion of task.

**Figure 3:**
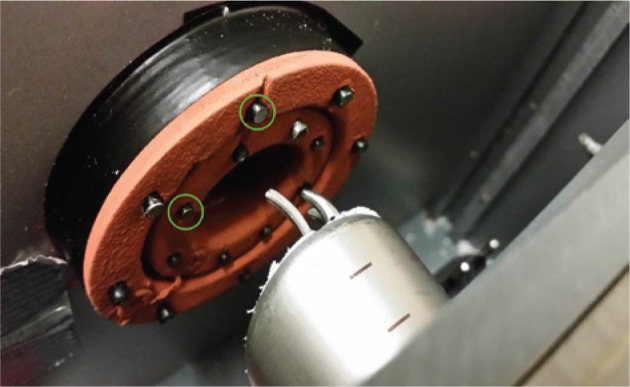
Cranial view on module 2 with introduced TEO^®^ system and instruments; green circle indicating targets.

**Figure 4:**
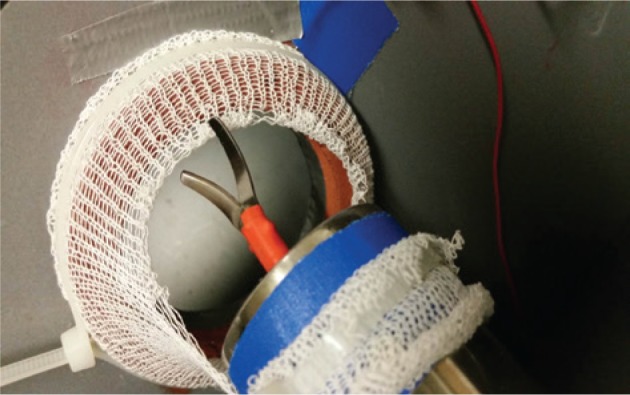
Cranial view on module 3 at half completion of the task.

Based on this catalog a modular box trainer was developed. As the NOTES platform, the TEO^®^ system (Karl Storz GmbH & Co. KG, Tuttlingen, Germany) was used. A box trainer containing three modules was developed with the modules being aligned in a linear manner (Figure 1). The first key step is simulated by module 3 (Figure 2). The area for PSS application is simulated by six pairs of steel loops. A needle and thread (PROLENE, Ethicon – Johnson & Johnson Medical GmbH, Norderstedt, Germany) must be passed through the pairs of loops clockwise or counterclockwise depending on the participant’s preference. The second step is simulated by module 1 (Figure 3). The task consists of contacting predefined spots with a laparoscopic hook. Contact outside the predefined area is electronically counted as an error (Figure 1). The third step is simulated by module 2 (Figure 4). The task demands cutting of a bandage within a predefined zone. Cutting outside this threshold leads to electric contact of the cutting instrument with the training device and is electronically counted as an error.

Assessment of the box trainer’s modules 1–3 was carried out by a cohort of medical students. To reduce bias by inter-participant differences in laparoscopic experience only laparoscopically naive students qualified for recruitment. All potential participants were scanned for any experience in laparoscopic procedures. Experience was defined as active or passive attendance of a single laparoscopic surgery. All students qualified for inclusion performed tasks 1–3 on the taTME-trainer. For all tasks, time for completion was measured. Additionally, in tasks 1 and 2 the number of prior defined errors was assessed.

All participants had to complete each task 5 consecutive times. The order of tasks was not as performed during taTME but instead in order of technical complexity starting with module 1 and finishing with the completion of module 3. Before processing each task, an instructional text was handed out to each participant and no further information was given to reduce bias. Likewise, to reduce problems caused by bi-instrumental handling of the needle, a short preliminary practical introduction for needle handling was held before executing task 3. The learning effect was defined as significant improvement of performance between repetition 1 (R1) and 5 (R5).

Data was analyzed using SPSS 24 (IBM, Armonk, NY, USA). To test for Gaussian distribution the Kolmogorov-Smirnov test was performed. The mean was described for data following Gaussian distribution and the median for non-Gaussian distribution. A test for significance was carried out using the Wilcoxon signed-rank test and p<0.05 was defined as the threshold for significance. The effect size was described by Pearson’s correlation coefficient.

## Results

**Figure 5:**
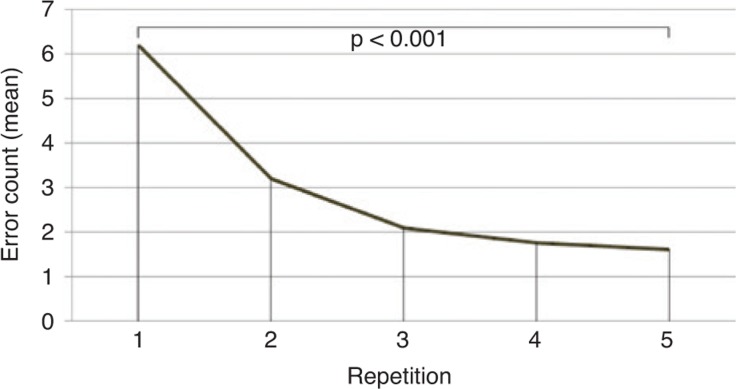
Error count over repetitions for task 1.

A learning effect for the first three tasks could be demonstrated. For task 1, a significant reduction in performance time (R1 vs. R5: p<0.001; Figure 5) as well as the number of errors (R1 vs. R5: p<0.001) was shown. The effect size was strong with Pearson’s correlation coefficient r=0.88 and 0.74, respectively. For task 2, a significant reduction in performance time (R1 vs. R5: p<0.001) as well as the number of errors (R1 vs. R5: p=0.001; Figure 5) was shown, analogously. Again, the effect size was strong with (r=0.86 and 0.74, respectively). For task 3 recording of errors was not executed. A significant reduction in processing time was shown (R1 vs. R5: p=0.001). The demonstrated effect size was strong (r=0.74). For all modules effect sizes decreased when comparing R4 with R5, indicating a learning effect.

## Discussion

Laparoscopic training has evolved to become a keystone of surgical apprenticeship. Training models are manifold and vary from virtual reality (VR) to ex-vivo box trainers and living animal training models [27]. While most realistic scenarios are obtained in living animal models or human cadaver experiments these training setups require much effort as well as a dedicated infrastructure. Technical progress has made VR training an attractive alternative for basic training, but asset costs are high and limit availability to large centers. Transferability of learned content into surgical reality is limited by lack of haptic feedback and any outside-the-box steps [27], [28], [29], [30].

Training models for NOTES scenarios exist, but a dedicated taTME box training has not yet been established [31]. While other training models focus on general competencies in minimally-invasive surgery the taTME-trainer is designed for specifically training proficiencies needed for taTME by simulating the key steps of these procedures. The device aims to be a tool for comparison and assessment of platforms and instruments as well as a tool for training.

As in laparoscopic box training in general, manifold limitations for transfer into surgical practice exist. The taTME-trainer presents abstracted tasks for training and device assessment. At best, the box trainer can achieve similarity to real surgical tasks. Similarity, in this case, is defined as similarity of performed movements and requirements for the surgeon. We adjusted the order of tasks to generate an increase of complexity. While the first task focuses on working with one instrument, use of the NOTES platform and acquisition of perception of depth, the following tasks combine the necessity of using a second instrument with more complex handling. This adjustment was chosen to reduce excessive demands for the laparoscopy naive participants. Significant differences between R1 and R5 indicate a learning curve. While manifold techniques for measurement of learning curves and learning effect have been described, the procedure time has been repeatedly taken into account for assessment of surgical learning effects [32], [33].

The taTME-trainer can be considered to train (basic) skills needed for transanal surgery. Technical challenges can be encountered and repeatedly trained before training on animals or human cadaver setups. Moreover, single- and multi-use platforms can be evaluated for suitability for transanal access and instrument movement. Therefore, taTME-trainer might reduce the use of animals and add to the efforts of surgical education in this niche of minimally-invasive access surgery.

## Supplementary Material

The article (iss-2019-0013) offers reviewer assessments as supplementary material.
