# Supplementary material for: Novel box trainer for taTME – prospective evaluation among medical students
Source: Innov Surg Sci. 2019 Oct 7;4(3):116–20. doi: 10.1515/iss-2019-0013 (PMC6817727; doi:10.1515/iss-2019-0013)
Supplement: Supplementary file 1 [file iss-4-20190013-s001.pdf]

Reviewer Assessment

Jakob Mann, Jens Rolinger, Steffen Axt, Andreas Kirschniak* and Peter Wilhelm

Novel box trainer for taTME – prospective evaluation among medical students

https://doi.org/10.1515/iss-2019-0013
Received July 29, 2019; accepted September 12, 2019

***Corresponding author: Andreas Kirschniak,** MD, MBA, Department of Surgery and Transplantation, University of Tuebingen, Hoppe-Seyler-Str. 3, 72076 Tuebingen, Germany, Phone: +49-(0)-7071/29-81233, E-mail: andreas.kirschniak@med.uni-tuebingen.de

Reviewers' Comments to Original Submission

Reviewer 1: Dr Timm Franzke

Aug 22, 2019

**Reviewer Recommendation Term:**	**Accept**
**Overall Reviewer Manuscript Rating:**	**N/A**
Is the subject area appropriate for you?	4
Does the title clearly reflect the paper's content?	4
Does the abstract clearly reflect the paper's content?	4
Do the keywords clearly reflect the paper's content?	5 - High/Yes
Does the introduction present the problem clearly?	4
Are the results/conclusions justified?	3
How comprehensive and up-to-date is the subject matter presented?	4
How adequate is the data presentation?	3
Are units and terminology used correctly?	5 - High/Yes
Is the number of cases adequate?	4
Are the experimental methods/clinical studies adequate?	4
Is the length appropriate in relation to the content?	5 - High/Yes
Does the reader get new insights from the article?	3
Please rate the practical significance.	3
Please rate the accuracy of methods.	4
Please rate the statistical evaluation and quality control.	4
Please rate the appropriateness of the figures and tables.	4
Please rate the appropriateness of the references.	5 - High/Yes
Please evaluate the writing style and use of language.	5 - High/Yes
Please judge the overall scientific quality of the manuscript.	4
Are you willing to review the revision of this manuscript?	Yes

Comments to Author:
The present work highlights an important topic, namely the further education of surgeons taking into account economic resources. The approach to decompose a complex operation into substeps is basically correct. An improved performance of the students could be achieved through the training. This is not surprising. Whether the box actually represents the partial steps of the taTME and whether this can actually lead to a shortening of the learning curve can not be answered on the basis of the study. Comparative studies with control groups would be necessary.

Open Access. © 2019 Mann J., et al., published by De Gruyter. This work is licensed under the Creative Commons Attribution 4.0 Public License.

Reviewer 2: anonymous

Jun 23, 2019

**Reviewer Recommendation Term:**	**Accept**
**Overall Reviewer Manuscript Rating:**	**70**
Is the subject area appropriate for you?	4
Does the title clearly reflect the paper's content?	4
Does the abstract clearly reflect the paper's content?	4
Do the keywords clearly reflect the paper's content?	5 - High/Yes
Does the introduction present the problem clearly?	4
Are the results/conclusions justified?	3
How comprehensive and up-to-date is the subject matter presented?	4
How adequate is the data presentation?	3
Are units and terminology used correctly?	5 - High/Yes
Is the number of cases adequate?	4
Are the experimental methods/clinical studies adequate?	4
Is the length appropriate in relation to the content?	5 - High/Yes
Does the reader get new insights from the article?	3
Please rate the practical significance.	3
Please rate the accuracy of methods.	4
Please rate the statistical evaluation and quality control.	4
Please rate the appropriateness of the figures and tables.	4
Please rate the appropriateness of the references.	5 - High/Yes
Please evaluate the writing style and use of language.	5 - High/Yes
Please judge the overall scientific quality of the manuscript.	4
Are you willing to review the revision of this manuscript?	Yes

Comments to Author:

The author presents in this paper an easy method for initial taTME-training analogous to other training skills in minimal invasive surgery with demonstration of a significant but expected learning effect. This kind of simulator might help to train working in a narrow space and gaining first experience in trananal operations with reduced triangulation but cannot compensate the need for enhanced training under widely real conditions. Therefore digital training forms like virtual reality might be more appropriate for beginning as well as advanced rectal surgeons to get further experience in this Special technique with its difficult learning curve.
